# Classic Unprovoked Takotsubo Syndrome: A Case Report

**DOI:** 10.7759/cureus.36056

**Published:** 2023-03-13

**Authors:** Abhirami Shankar, Narayanaiyengar Devaraj

**Affiliations:** 1 Internal Medicine, West Anaheim Medical Center, Anaheim, USA; 2 Cardiology, West Anaheim Medical Center, Anaheim, USA

**Keywords:** chf, heart failure, troponin, myocardial infarction, acs, nstemi, stemi, coronary, cardiomyopathy, takotsubo

## Abstract

Although Takotsubo syndrome (TS) has been long recognized, it is now more frequently identified as a cause of stress-induced cardiac injury since its first description in the 1990s. While most cases are transient, many patients can have acute and long-term effects including persistent or worsening heart failure, arrhythmia, cardiac thrombi, outflow tract obstruction, ventricular wall rupture, and cardiogenic shock. Medical optimization is necessary to prevent cardiac remodeling and disease recurrence and manage associated heart failure. The choice of medications may vary from patient to patient based on the inciting factor or the most probable cause. Anticoagulation can be added for a small period of time if there is a concern for thrombus formation from akinesia/dyskinesia. Most patients achieve early recovery and resolution of symptoms and those with persistent manifestations can be managed medically.

## Introduction

Takotsubo syndrome (TS) is a cardiac disorder that can cause transient wall motion abnormalities (WMA), and hypokinesia/dyskinesia/akinesia of segments of the heart, which determines the “type” of Takotsubo cardiomyopathy (apical, focal, etc.) [1]. The classic form causes apical ballooning with WMA in mid and apical regions [1]. TS can be primary or secondary based on whether it is the cause of initial presenting symptoms, a result of hospitalization or surgery, or caused by another disease like thyrotoxicosis [1]. Generally, it is considered transient with favorable long-term outcomes. However, patients still need to be on medical therapy depending on the cause and clinical course.

## Case presentation

A 58-year-old female presented due to a first-time episode of dull retrosternal chest pain, tightness, and pressure with exertion and at rest, rated 10/10 in intensity, radiating to the back in the left interscapular region since that morning lasting about 1.5 hours. She endorsed profuse diaphoresis, dyspnea, lightheadedness, and palpitations but denied syncope and loss of consciousness. She had experienced a similar episode many years ago but had never consulted a cardiologist. She was compliant with her medications including losartan 25 mg daily and levothyroxine 88 mcg daily. She denied vaping or alcohol and illicit drug use. Her smoking status was unknown (she possibly smoked a few cigarettes daily for 10 years). The physical exam was unremarkable except for mild chest tenderness.

**Figure 1:**
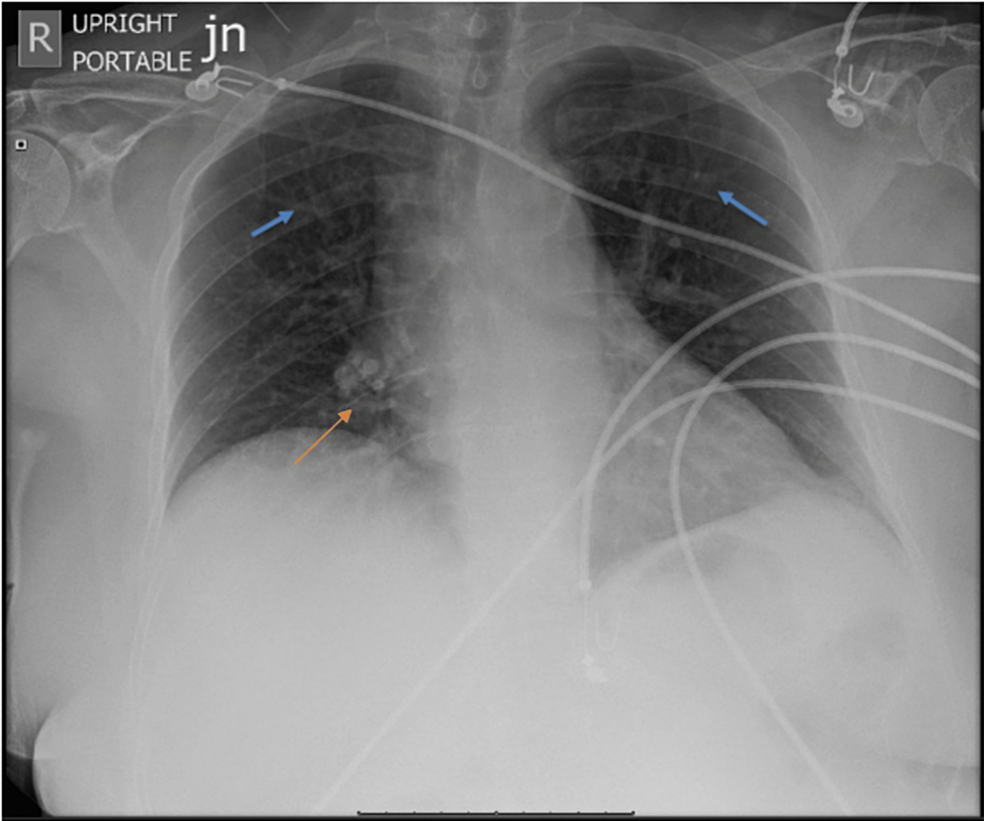
Chest X-ray showing a normal cardiac silhouette Some calcification is present in the right hilar region (orange arrow) and aortic knob. There is a slight prominence of vascular markings (blue arrows)

The patient's past medical and surgical history included hypertension, hyperlipidemia, hypothyroidism, and meningioma s/p craniotomy ~8 years ago. She denied any history of acute coronary syndrome/myocardial infarction (ACS/MI), congestive heart failure (CHF), heart murmur, rheumatic fever, arrhythmia, heart block, and anxiety. In pertinent family history, her father had experienced a stroke. Differential diagnoses included non-ST-elevation myocardial infarction (NSTEMI), pulmonary embolism, aortic dissection, pneumothorax, pericarditis, pericardial tamponade, esophageal rupture, pneumonia, pleurisy, gastroesophageal reflux disease (GERD), and musculoskeletal chest pain. Her heart score was 5. Chest X-ray was unremarkable except for mild cephalization (Figure 1).

**Figure 2:**
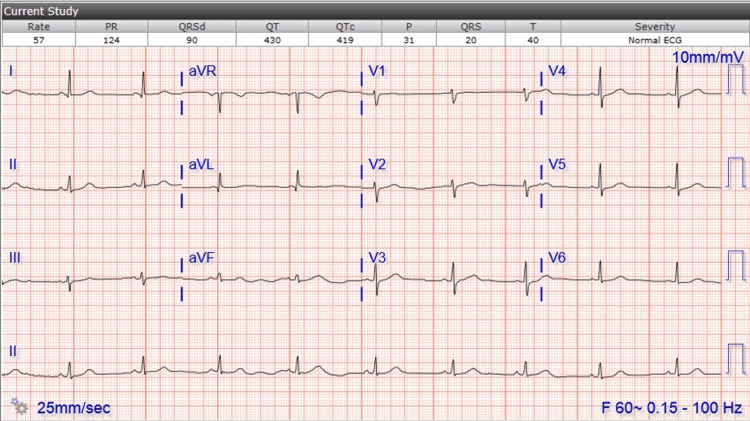
EKG on admission: sinus bradycardia HR 57, no ST elevation, no ectopy. Small r-wave in V1 and V2, abnormal T/T-wave variation anterior leads, small q-wave in I EKG: electrocardiogram

Vitals showed a temperature of 97.7 °F, a heart rate of 64 beats per minute, and a respiratory rate of 15 breaths per minute. Her blood pressure was 112/76 mmHg. Labs were significant for elevated ALT (~39), leukocytosis (~11.2K), low thyroid stimulating hormone (TSH)/free T4 (~0.182/0.61), and elevated triglycerides (~258). UDS, coagulation panel, electrolytes, D-dimer, and SARS tests were unremarkable. HS troponin trend was as follows: admission: 207→2794→1478→; day of cath: 573→2148→1974→; POD 1: 1199→712→642→; POD 2: 616→544. Figure 2 shows the EKG on admission.

**Figure 3:**
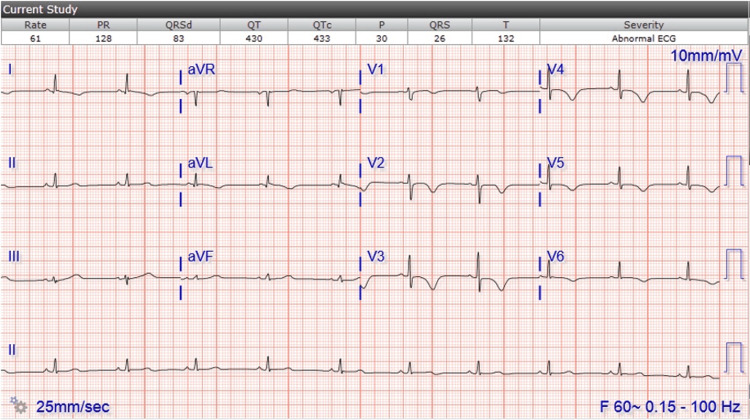
EKG on the day of cath: sinus bradycardia with T-wave inversion across the precordial leads, lead I and aVL Findings are consistent with anterolateral ischemia and/or nontransmural injury EKG: electrocardiogram

**Figure 4:**
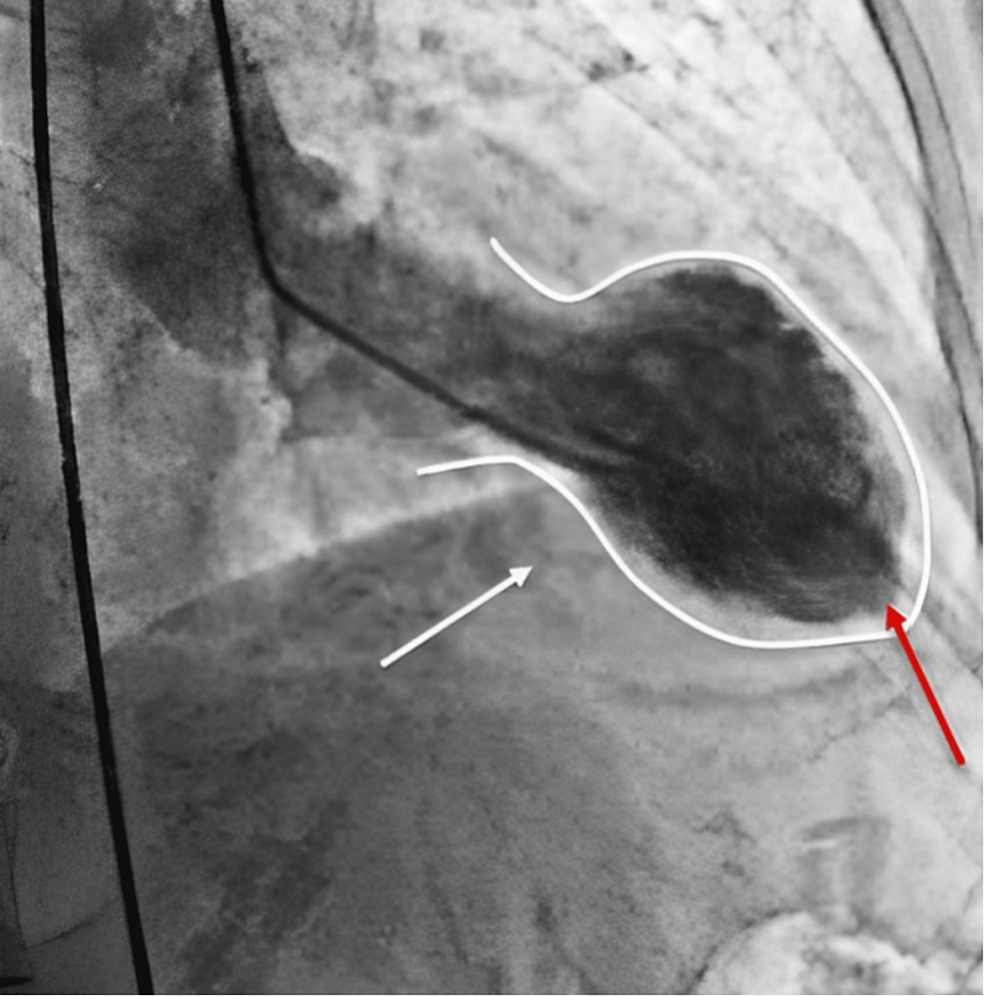
Cardiac catheterization image showing Takotsubo heart (white arrow and outline). Type: apical ballooning with “nipple sign” (red arrow)

The patient was given fluids, aspirin, and nitroglycerin and admitted to telemetry. Cardiology was consulted. The patient’s chest pain initially subsided with the above but recurred as dull chest pressure. She was otherwise hemodynamically stable. Troponin was elevated and repeat EKG showed T-wave inversions not present on the initial EKG (Figures 2, 3). A full dose of Lovenox was initiated. Echo (Table 1) showed WMA. She had cardiac catheterization with fluoroscopy supervision of the left heart and coronaries (Table 2) for NSTEMI, which showed Takotsubo cardiomyopathy (Figure 4).

**Table 1:** Echocardiogram findings prior to cardiac cath LVEF: left ventricular ejection fraction; RVSP: right ventricular systolic pressure; AoV: aortic valve; Vel: velocity; AO: aortic; Gr: gradient; MV: mitral valve; TR: tricuspid; RAP: right atrial pressure; PV: pulmonic valve; IVC: inferior vena cava

Location	Findings
Left ventricle	Normal size. Systolic function is relatively preserved. Hypertensive heart disease. Significant regional wall motion abnormalities are noted (severe hypokinesis of distal septum and apex). The left ventricular diastolic function is normal. LVEF is 50-55%
Right ventricle	Normal size. Systolic function is normal. RVSP 13 mmHg
Left and right atria	Normal size
Aortic valve	Trileaflet. Mild valve sclerosis. No regurgitation. AoV peak Vel. 115.2 cm/s. AO peak Gr. 5.3 mmHg
Mitral valve	Mild annular calcification. Mild regurgitation. MV E Vel. 79.7 cm/s. MV peak Gr. 26 mmHg. MV DECEL time 144 ms. MV A Vel. 66.5 cm/s. E/A ratio 1.2
Tricuspid valve	Normal structure. Trace regurgitation. TR peak Vel. 90 cm/s. RAP estimate 10 mmHg. TR peak Gr. 3 mmHg
Pulmonic valve	Normal structure. Mild regurgitation. PV peak Vel. 67.5 cm/s. PV peak Gr. 2 mmHg
Great vessels	The aortic root is of normal size. IVC is normal in size and collapses ~50% with inspiration
Pericardium	No pericardial/pleural effusion

**Table 2:** Angiography findings

Region	Angiographic findings
Left ventricular cineangiogram performed in 30 degrees right anterior oblique projection	Apical hypokinetic and akinetic area consistent with Takotsubo cardiomyopathy
Left main coronary artery	Widely patent
Left anterior descending coronary artery	Transapical vessel, widely patent with no arteriosclerotic changes
Circumflex artery	Non-dominant vessel, widely patent with no arteriosclerotic changes
Right coronary artery	Large dominant vessel, widely patent with no arteriosclerotic changes
Right iliofemoral angiography	Patent common iliac and common femoral artery. The catheter entry point is well above the bifurcation

The patient was discharged in stable condition and advised to follow up with the cardiologist as an outpatient. She was discharged home on aspirin, atorvastatin, lisinopril, and metoprolol succinate. She was subsequently readmitted for orthopnea and midsternal, non-radiating, reproducible pleuritic chest pain. Her troponin remained elevated and was likely secondary to demand ischemia, periprocedural myocardial injury, and LV dysfunction. Repeat limited Echo showed EF of ~55% but with no significant WMA as seen on the previous Echo. Cardiologist readjusted her medications with the addition of isosorbide mononitrate, furosemide, and spironolactone to address LV failure and to reverse remodeling, given her symptomatic presentation although EF was preserved. The patient’s symptoms resolved and she was discharged when stable. She has not had any further ER visits or readmissions to this hospital since.

## Discussion

Management of TS mainly involves supportive therapy. Approach to acute management can be based on the suspected underlying cause, although this is difficult to achieve as patients presenting are usually worked up for other diseases such as acute MI, and the diagnosis is not made until after catheterization and left ventriculogram. Therapy corresponding to pathophysiology would mean beta-blockers in suspected autonomic hyperactivity, nitrates or calcium channel blockers in coronary vasospasm, etc. [2]. Heart failure can occur, especially in patients aged >70 years, and those with LVEF <40% and physical stressors [2]. Mild cases (stable vitals, minimal troponin leak, no cardiogenic shock) can be treated with classic heart failure guideline-directed therapy including ACE-I/ARB/ARNI, beta-blockers, mineralocorticoid receptor antagonists, and/or another diuretic. Some studies in the literature recommend initial treatment with the above along with dual antiplatelet therapy, anticoagulation, and statins [3]. If patients are asymptomatic and hemodynamically stable, pharmacotherapy should be carefully chosen to avoid inflicting iatrogenic harm on an otherwise naturally recovering process [2].

Short-course anticoagulation can be added especially if there is a concern for thrombus formation related to cardiac akinesia/dyskinesia with ballooning [2]. Patients with large areas of hypo/akinesia and/or LVEF <30% need to be observed more closely for heart failure, atrial/ventricular arrhythmias, cardiac thrombi, and cardiogenic shock [2]. Management of shock depends on the presence of left ventricular outflow tract obstruction (LVOTO); if LVOTO is present, inotropic agents are contraindicated and medications to increase diastolic filling time, such as IV metoprolol, esmolol, or landiolol, are recommended [2-3]. Arrhythmias due to QTc prolongation are also seen, but no prophylactic treatment is indicated for the same [4]. However, administering magnesium helps with acute phase TS-associated ventricular tachycardia with long QTc [4].

## Conclusions

Although TS is seen as a benign transient condition, it can lead to many acute complications. Some of these complications include acute heart failure, LVOTO, RV involvement, mitral regurgitation, arrhythmias, cardiac thrombus formation, pericardial effusion, ventricular wall rupture, and cardiogenic shock. Depending on the clinical analysis, hemodynamic stability, Echo findings, suspected diagnosis, and underlying pathophysiology, therapy needs to be tailored to individual patients. Mortality usually occurs due to refractory shock or fatal arrhythmias such as ventricular fibrillation. Data on long-term prognosis is limited; while most patients recover within a few weeks, there have been reports on subsets of patients who experience persistent symptoms such as exertional dyspnea, palpitations, and angina. Beta-blockers can exacerbate bradyarrhythmia or AV blocks, and hence they need to be used with caution. Inotropic agents can help in cardiogenic shock but are contraindicated in LVOTO. Thus, medical optimization is important to prevent future recurrence and long-term complications of the disease.
